# ENDOCRINOLOGY IN PREGNANCY: Targeting metabolic health promotion to optimise maternal and offspring health

**DOI:** 10.1530/EJE-21-1046

**Published:** 2022-04-05

**Authors:** Niamh-Maire McLennan, Jonathan Hazlehurst, Shakila Thangaratinam, Rebecca M Reynolds

**Affiliations:** 1 MRC Centre for Reproductive Health, University of Edinburgh, Queen’s Medical Research Institute, Edinburgh, UK; 2 Department of Diabetes and Endocrinology, University Hospital Birmingham Foundation Trust, Birmingham, UK; 3 WHO Collaborating Centre for Women’s Health, Institute of Metabolism and Systems Research, University of Birmingham, Birmingham, UK; 4 Birmingham Women’s and Children’s NHS Trust, Birmingham, UK; 5 BHF/University Centre for Cardiovascular Science, University of Edinburgh, Queen’s Medical Research Institute, Edinburgh, UK

## Abstract

There is an increase in maternal metabolic burden due to the rise in pregnancies complicated by obesity, gestational diabetes, type 2 diabetes and polycystic ovary syndrome. Metabolic dysfunction during pregnancy is associated with increased risks of long-term morbidity and mortality for women and their offspring. Lifestyle interventions in pregnancy in women at risk of metabolic dysfunction have demonstrated short-term improvements such as reduced gestational weight gain and lowered risk of gestational diabetes. It is not known whether these interventions lead to sustained improvements in the metabolic health of the mother and baby. Pharmacological interventions have also shown benefits for the mother and baby in pregnancy, including improvements in glycaemic control, reduction in gestational weight gain and reduction in large for gestational age infants; however, there remains uncertainty over long-term outcomes for mother and child. Existing studies on interventions targeting metabolic health are limited to selected populations in the preconception and postpartum periods and lack follow-up beyond delivery of the intervention. The COVID-19 pandemic has refocused our attention on the effects of maternal metabolic ill-health that play a role in contributing to premature morbidity and mortality. There is an urgent need for strategies to accurately identify the growing number of women and offspring at risk of long-term adverse metabolic health. Strategies which focus on early identification and risk stratification using individualised risk scores in the pre and inter-conception periods must take priority if we are to target and improve the metabolic health of women and their offspring who are at highest risk.

## Invited Author’s profile


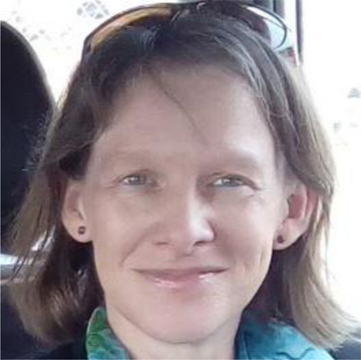


**Prof. Reynolds** is Dean International, College of Medicine and Veterinary Medicine, University of Edinburgh, Prof. of Metabolic Medicine, University of Edinburgh, Honorary Consultant Physician in Diabetes and Endocrinology, NHS Lothian and Deputy Head of the Centre for Cardiovascular Sciences, University of Edinburgh. Her research focus is a life-course approach to the prevention of non-communicable diseases with a particular interest in exposures in pregnancy including maternal obesity, gestational diabetes and glucocorticoid hormones. She has (a) used healthcare record data-linkage and cohort studies to document the consequences of a mother’s health in pregnancy on the health of next and future generations; (b) identified underpinning mechanisms through experimental medicine studies embedded within clinical practice and (c) tested novel interventions to improve pregnancy outcomes in clinical trials. She is currently extending this work by leading an MRC-funded study to set up a new pregnancy cohort ‘Born in Scotland in the 2020s’.

## Overview

## Burden of the problem

### Maternal obesity and hyperglycaemia

Obesity (BMI > 30 kg/m^2^) is a chronic complex disease. Figures from 2019 suggest that 17% of adult women living in European countries have a BMI of >30 kg/m^2^ and 46% are classified overweight (BMI > 25 kg/m^2^) (https://ec.europa.eu/eurostat/statistics-explained/index.php?title=Overweight_and_obesity_-_BMI_statistics#Obesity_in_the_EU:_gender_differences, accessed 29 January 2022). Obesity prevalence at antenatal booking has more than doubled in a decade (1). Currently, 22% of pregnant women have obesity recorded at their first antenatal review (2).

Obesity is a principal driver for metabolic dysfunction in pregnancy, particularly gestational diabetes (GDM) and type 2 diabetes. A pan European study identified the prevalence of GDM among overweight/obese women to be 39% (3). Similarly, in the USA, pregnant women with obesity have a nearly four-fold increased risk of developing GDM compared to pregnant women of healthy weight, rising to eight-fold increased risk in those with a BMI > 40 kg/m^2^ (4). Rising rates of GDM over the past two decades are attributed to both the obesity epidemic as well as changes in diagnostic criteria (5) with prevalence as high as 25% depending on the population studied. GDM is a well-established risk factor for subsequent type 2 diabetes across the maternal life course. The increasing rates of pre-existing type 2 diabetes in women entering pregnancy are of significant concern. A national audit in England and Wales in 2019 demonstrated a 28% increase in maternal type 2 diabetes over 5 years (6), while prevalence in Canada has doubled over the last 20 years and in the USA, a 4-fold increase was reported between 1994 and 2014 (7).

### Effects of metabolic dysfunction on mother and offspring

Maternal obesity and GDM both have independent and additive effects on adverse maternal and neonatal outcomes. Stillbirth, perinatal death, neonatal death and infant mortality all increase across BMI categories (8). The long-term risk to the offspring persists into adulthood. Compared to offspring born to lean women, those born to obese women are more likely to be obese in childhood and adulthood (9, 10, 11). Elevated maternal BMI is associated with an increased risk of cardiovascular disease, stroke, type 2 diabetes mellitus (statistically significant in females only) and premature all-cause mortality in adult offspring (12). A follow-up of the offspring born to women with GDM, at 10–14 years of age, demonstrated childhood impaired glucose tolerance and childhood adiposity, independent of maternal BMI (13, 14). For the mother, short-term complications include an increased risk of GDM, hypertensive disorders of pregnancy and intrapartum intervention (5). Babies born to mothers with GDM and/or obesity are at risk of large for gestational age (fetal weight > 90th centile) or macrosomia (>4kg), consequently leading to increases in birth-related injuries including shoulder dystocia, hypoxic brain injury, fractures and nerve palsies (5). Women with type 2 diabetes during pregnancy are at higher risk of congenital malformations and of still birth (23 per 1000 births (95% CI: 16.4, 31.8)) (6, 15).

Long-term follow-up studies demonstrate women with metabolic dysfunction during pregnancy are at risk of future cardiometabolic disease’. A systematic review, including 20 studies, and 675 455 women with GDM, conferred a relative risk (RR) of 7.42 (95% CI: 4.79, 11.51) of developing type 2 diabetes, representing a seven-fold increased risk over women without GDM (16). A systematic review including 5 390 591 women demonstrated a two-fold higher RR of 1.98 (95% CI: 1.57, 2.50) of future cardiovascular events in women with GDM and identified this doubling of risk was independent of onset of type 2 diabetes (17).

In this review, we collate evidence on the identification of women at risk of metabolic ill-health during pregnancy and review interventions which target improving maternal and offspring metabolic health. Presented data include long-term metabolic health of mother and offspring where available.

## Early identification of women in pre- and early pregnancy with diseases contributing to maternal and offspring metabolic risk

Obesity, polycystic ovary syndrome (PCOS), GDM and pre-existing type 2 diabetes all predispose both the mother and offspring to metabolic risk, though the extent to which their early identification can lead to interventions that modify this risk is less certain.

### Obesity

The identification and treatment of women with obesity are recommended pre-pregnancy and given the low uptake and availability of pre-conception services, it is recommended that this forms a part of an opportunistic health strategy embedded within other clinical appointments (18, 19). Clinicians often feel unprepared to have these conversations, though the modified ‘5 As’ approach (Ask, Assess, Advise, Agree, Assist) provides a usable framework for initial weight management assessment by a non-obesity specialist (Table 1) (20).

**Table 1:** Modified 5 As approach for weight management assessment. Adapted from obesitycanada.ca/resources/5as/.

**Ask** for permission to discuss weight and explore readiness
**Assess** obesity-related risks and ‘root causes of obesity’
**Advise** on health risks and treatment options
**Agree** on health outcomes and behavioural goals
**Assist** on accessing appropriate resources and providers

The overly simplistic classification of obesity risk by BMI is highlighted by the fact that over half of pregnancies in women with obesity proceed without complication if the expectant mother has no pre-existing medical conditions or early obstetric complication (21). More accurate methodologies are required to identify risk in patients with obesity to target those most at risk and avoid over medicalisation of all obese women.

### Polycystic ovary syndrome (PCOS)

Given the association between obesity and PCOS, it is important to recognise that PCOS is an independent risk factor for adverse delivery and neonatal outcomes, as has been shown by a large retrospective study of >9 million births (22). The hyperandrogenic phenotype appears associated with the most increased maternal risk. A multicentre study in the Netherlands demonstrated an increased risk of GDM in women with PCOS compared to a reference group of women, adjusted odds ratio (OR) of 4.15 (95% CI: 2.07, 8.33). Subgroup analysis of women with PCOS identified a heightened risk of GDM in the hyperandrogenic group, compared to a reference group of women, adjusted (OR) for hyperandrogenic women was 5.65 (95% CI: 2.49, 12.81) and for the normoandrogenic PCOS women adjusted OR was 3.17 (95% CI: 1.28, 7.84) (23).

### Gestational diabetes mellitus

Previous GDM is the strongest predictor of GDM in a subsequent pregnancy (24), with recurrent GDM rates reported as high as 84%, depending on population and diagnostic criteria (25). Several international guidelines for the management of GDM recommend self-monitoring of blood glucose or an oral glucose tolerance test (OGTT) as soon as possible for women with previous GDM who book in the first trimester (26, 27, 28). However, in a study conducted in the UK, almost half of all women with identified risk factors fail to have an OGTT performed in routine care settings (https://www.npeu.ox.ac.uk/assets/downloads/mbrrace-uk/reports/maternal-report-2020/MBRRACE-UK_Maternal_Report_Dec_2020_v10_ONLINE_VERSION_1404.pdf, accessed 25rd July 2021). Groups least likely to undergo an OGTT include women from ethnic minority backgrounds, women with obesity and those with a family history of diabetes (29). Women who are at risk, but unscreened, face worse pregnancy outcomes including a 44% increase in still birth compared to low-risk women (30). These data demonstrate the need to improve uptake of screening and to prioritise early diagnosis of GDM in high-risk patient groups.

### Pre-existing diabetes

Women with pre-existing diabetes are encouraged to seek pre-pregnancy counselling. UK-based National Institute for Health and Care Excellence (NICE) recommends targeting an HbA1c < 48 mmol/mol pre-pregnancy, as long as it is not at the expense of significant hypoglycaemia. NICE further recommends avoiding pregnancy if HbA1c > 83 mmol/mol (https://www.nice.org.uk/guidance/ng3/resources/diabetes-in-pregnancy-management-from-preconception-to-the-postnatal-period-pdf-51038446021, accessed 23rd July 2021). In addition to the glycaemic targets, risk factor modification and adjustment of medications, folic acid supplementation and weight management are important parts of pre-conception counselling. Despite the guidance, the National Diabetes in Pregnancy Audit highlighted that in England, >80% of women with type 1 diabetes and >60% of women with type 2 diabetes have a first-trimester HbA1c outside of these targets (6). Deprivation and obesity are consistently associated with HbA1c > 48 mmol/mol in both women with type 1 and type 2 diabetes. Given the increasing availability of continuous glucose monitoring in pregnancy for women with type 1 diabetes, it is hoped that there will be further improvement in glycaemic control prior to and during pregnancy.

### Screening and prediction of metabolic risk

Apart from screening criteria for GDM and the pre- and intra- pregnancy care of women with pre-existing diabetes, there are no established screening mechanisms for women at increased risk because of other metabolic comorbidities (26). PCOS, in particular, does not trigger screening for GDM in most guidance, despite the proven increased risk (22).

Half of the women with pregnancies complicated by GDM will develop type 2 diabetes within 5 years of the index pregnancy. Early identification and management of type 2 diabetes can lead to remission and ameliorate long-term cardio-metabolic health risks (31). With this knowledge, international and national bodies recommend post-partum screening strategies for women who have had GDM to identify type 2 diabetes at an early stage) (27, 32, 33, 34). However, adherence globally has been low; one systematic review in 2011 highlighted over a fifth (20–66%) of all women failed to complete any post-partum screening (35). In recent years, little improvement has been seen. A study published in 2018, examining electronic records from 127 primary care practices across England found less than a fifth of women who had experienced GDM had blood glucose testing 6 months after giving birth (36). Several healthcare-related factors are likely to contribute to these findings and include both patient and healthcare provider factors. Qualitative studies among healthcare providers have attempted to identify barriers to post-partum follow-up of women with GDM (37). Common themes identified include gaps between clinician knowledge regarding the risk of type 2 diabetes following GDM and practice of postpartum screening of these women. Other key themes identified are a lack of communication of the diagnosis of GDM among clinicians as well as clarity on responsibility for follow-up of women with GDM. There is a clear need to improve referral pathways and communication systems between GDM-related care providers and to ensure that clinicians are provided with adequate training on postpartum screening.

Currently, few prediction models for type 2 diabetes include GDM, and none of them account for pregnancy-specific characteristics. Well recognised predictors of type 2 diabetes in women with GDM include raised BMI RR of 1.95 (95% CI: 1.60, 2.31), family history of diabetes RR of 1.70 (95% CI: 1.47, 1.97), non-white ethnicity RR of 1.49 (95% CI: 1.14, 1.94) and advanced maternal age RR of 1.20 (95% CI: 1.09, 1.34). However, in addition to these well recognised predictors, early diagnosis of GDM RR of 2.13 (95% CI: 1.52, 3.56), raised fasting glucose RR of 3.57 (95% CI: 2.98, 4.04), increased HbA1c RR of 2.56 (95% CI: 2.00, 3.17) and use of insulin RR of 3.66 (95% CI: 2.78, 4.82) are also associated with an increased risk of subsequent type 2 diabetes. Other patient-specific factors which were found to predict future risk of type 2 diabetes were multiparity RR 1.23 (95% CI: 1.01, 1.50), hypertensive disorders in pregnancy RR 1.38 (95% CI: 1.32, 1.45) and preterm delivery RR 1.81 (95% CI: 1.35, 2.43) (38).

Ethnicity is a strong predictor of both GDM and Type 2 DM. Recently studies have identified lower BMI thresholds for non-white populations in predicting incidence of GDM and type 2 diabetes (39, 40). A Canadian study found the prevalence of GDM exceeded 5% at an estimated BMI of 21.5 kg/m^2^ among South Asian women, 23.0 kg/m^2^among Chinese women and 29.5 kg/m^2^ among the general population (40). Similarly, in a population-based cohort study (1 472 819 people) for the equivalent age-adjusted and sex-adjusted incidence of type 2 diabetes at a BMI of 30.0 kg/m² in White populations, the BMI cut-offs were 23.9 kg/m² (95% CI: 23.6–24.0) in South Asian populations, 28·1 kg/m² (28.0–28.4) in Black populations, 26.9 kg/m² (26.7–27.2) in Chinese populations, and 26.6 kg/m² (26.5–27.0) in Arab populations (39). Incorporating lower BMI thresholds for non-white women into pre-pregnancy, antenatal and postnatal care could improve identification of women at risk of short- and long-term metabolic dysfunction, triggering earlier-targeted lifestyle/dietary intervention.

**Figure 1:**
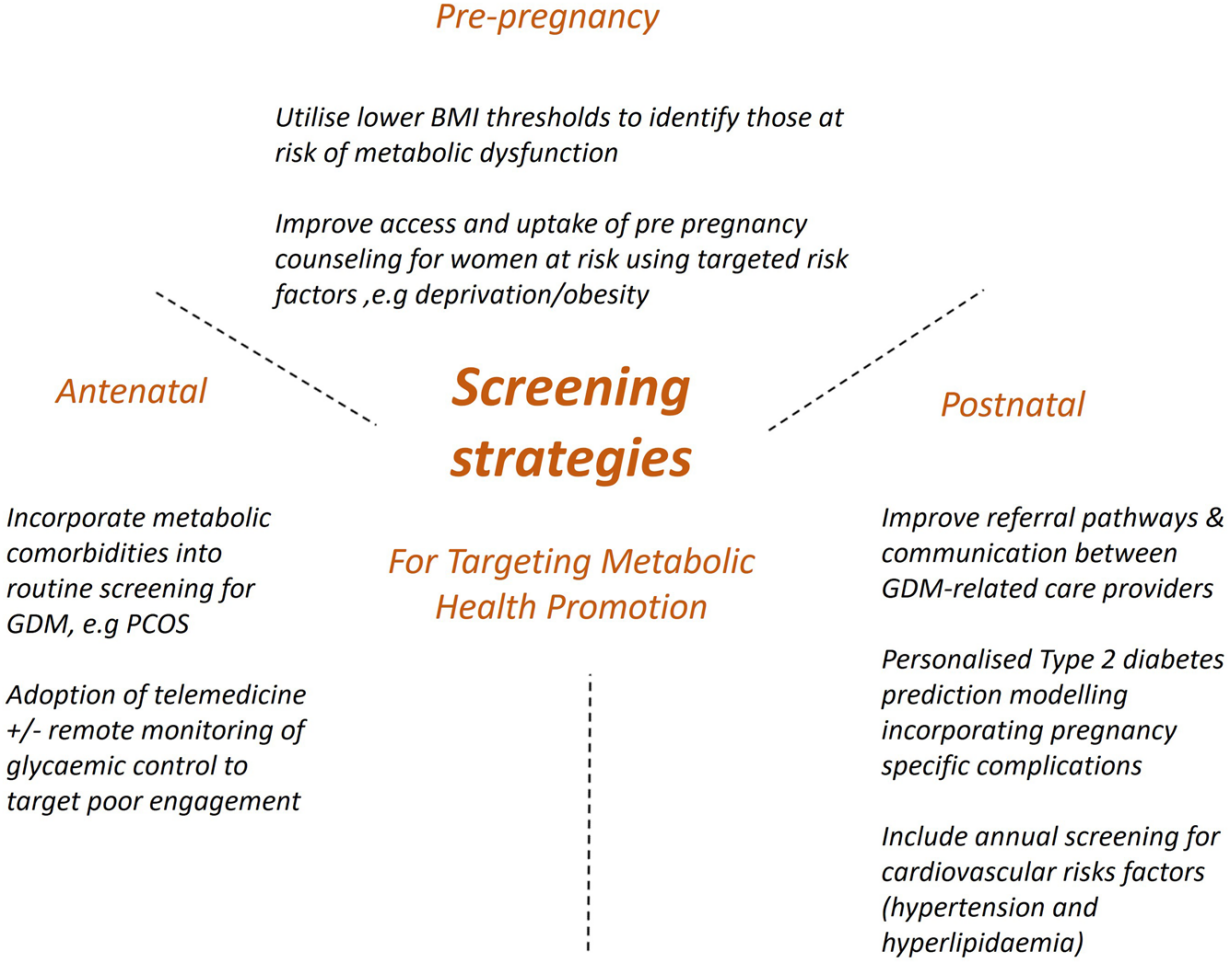
Screening strategies for targeting metabolic health promotion.

Screening for established risk factors, as well as prediction modelling of pregnancy complications, may allow more targeted intensive monitoring of at-risk women and preventative treatment, as well as potentially limiting the over treatment or investigation of comparatively lower-risk women, see Fig. 1 for a summary of screening strategies targeting metabolic health promotion. Furthermore, personalised risk communication with quantitative estimates can increase the number of individuals that make informed choices in screening programmes (11). Given the rising prevalence of GDM and the associated complications, there is significant ongoing work to establish a prediction model to identify complications in women with GDM (41).

## Interventions

Interventions to improve the long-term metabolic health of the offspring and mother, as well as to minimise pregnancy-related complications, should address the entire life course rather than be limited to the antenatal period (42).

### Interventions in pregnancy

#### Diet and physical activity

Considerable research has focused on dietary and physical activity-based interventions in pregnancy. Meta-analysis of aggregate data of diet and physical activity-based weight loss interventions has shown effects on gestational weight gain, though impact on pregnancy-specific outcomes has varied (43). Subgroup analysis showed a reduction in gestational weight gain in women with overweight and obesity, though this did not translate into changes in birthweight or pre-eclampsia (43). A meta-analysis of individual participant data (IPD) from 36 randomised trials (12 526 women) (44) reported less gestational weight gain in the intervention group than control group, with a mean difference of −0.70 kg (95% CI: −0.92, −0.48). Though not significant, the summary estimates favoured the intervention group for risk reduction of maternal and offspring composite outcomes, with no evidence that this effect differed across subgroups defined by maternal characteristics. Within the IPD meta-analysis, there was evidence for a reduction in caesarean section in the intervention groups but no other individual outcomes. When the IPD data were supplemented with study-level data, from studies not providing IPD, an effect was seen on rates of GDM (44). A further IPD meta-analysis of randomised trials is in progress with the aim of assessing the differential effects and cost-effectiveness of diet and physical activity-based interventions in preventing GDM and its complications (45).

There has been recent interest in the use of Mediterranean-style diets in pregnancy. A large, UK-based, multi-centre pragmatic randomised–controlled trial, Mediterranean-style diet in pregnant women with metabolic risk factors (ESTEEM), demonstrated a significant reduction in GDM risk by 35%, adjusted OR of 0.65 (95% CI: 0.47, 0.91) as well as a reduction in gestational weight gain, mean of 6.8 kg vs 8.3 kg; adjusted difference of −1.2 kg (95% CI: −2.2, −0.2) in women following a Mediterranean-style diet compared to usual care (46). The diet included high intake of nuts, extra virgin olive oil, fruit, vegetables, unrefined grains and legumes; moderate to high consumption of fish; low to moderate intake of poultry and dairy products such as yogurt and cheese; low consumption of red meat and processed meat and avoidance of sugary drinks, fast food and food rich in animal fat. Participants were provided with mixed nuts and extra virgin olive oil to increase their intake. The advice was delivered at 18, 20 and 28 weeks. Of the women randomised within the study, 60% were of Black or Asian ethnicity and 69% had obesity. Although this study was limited by a reliance on self-reported adherence to dietary intervention, it demonstrated a significant reduction in GDM risk and gestational weight gain (46). An additional study of Mediterranean diet in pregnancy (the St Carlos study) compared two groups given basic advice regarding a Mediterranean diet with the control group advised to restrict dietary fat and the intervention group provided with extra virgin olive oil and pistachio nuts with minimal daily consumption guidance. The RR for GDM was 0.75 (95% CI: 0.57, 0.98) in the intervention vs the control group (47). A meta-analysis of pooled data between the two studies showed a consistent reduction in the risk of GDM, OR of 0.67 (95% CI: 0.53, 0.84) (46).

#### Pharmacological

Pharmacological interventions for women with GDM target maternal insulin resistance. Two landmark, randomised trials (48, 49) demonstrated a reduction in birthweight and large‐for‐gestational‐age infants in women with GDM who received treatment (combination of dietary advice, self-monitoring of blood glucose and insulin therapy) compared with women with GDM who were not treated. This led to a plethora of studies investigating the potential benefits of various glucose-lowering agents and s.c. insulins (50). In a meta-analysis including 35 trials of pharmacological interventions for GDM, metformin was reported as an effective alternative to insulin in the treatment of hyperglycaemia; however, supplemental insulin may be required in up to 50% of women (51). A systematic review of neonatal outcomes in studies of metformin vs insulin included nine studies reporting measures of neonatal growth. Neonates born to metformin-treated mothers had significantly lower birth weight, with mean difference of −107.7 g (95% CI: −182.3, −32.7) and lower ponderal index and mean difference of −0.13 kg/m^3^ (95% CI: −0.26, 0.00) than neonates of insulin-treated mothers (52).

Emerging long-term follow-up studies have started to address whether optimising glycaemic control during pregnancy has any long-term metabolic health impact on women or their offspring. A systemic review and meta-analysis of neonatal, infant and childhood growth following metformin vs insulin treatment for GDM included 28 studies (52). Two studies (*n*  = 411 infants) reported measures of infant growth at 18–24 months of age. Metformin-exposed infants were significantly heavier than those in the insulin-exposed group, with a mean difference of 440 g (95% CI: 50, 830). Three studies (*n*  = 520 children) reported mid-childhood growth parameters (5–9 years). In mid-childhood, BMI was significantly higher, with a mean difference 0.78 kg/m^2^ (95% CI: 0.23, 1.33) following metformin exposure compared with insulin exposure, although the difference in absolute weights between the groups was not significantly different (*P*  = 0.09). While these data provide reassurance regarding the safety of metformin use during pregnancy, there is no clear evidence that exposure to this insulin-sensitising agent has lasting favourable effects on body composition in the offspring of women with GDM.

Pharmacotherapy is an established approach in the management of pre-existing type 2 diabetes that cannot be sufficiently controlled with diet alone. The metformin in type 2 diabetes (Mity) trial is the largest trial of metformin vs placebo in addition to standard regimen of insulin in 502 women with type 2 diabetes during pregnancy (53). No difference between groups was found in the primary outcome, a composite of serious neonatal outcomes. Compared with women in the placebo group, metformin-treated women achieved significantly better glycaemic control (HbA1c at 34 weeks’ gestation, 41.0 (s.d. 8.5) mmol/mol vs 43.2 (s.d. −10) mmol/mol) and had lower insulin requirements, 1.1 units per kg per day vs 1.5 units per kg per day; difference: −0.4 (95% CI: −0.5, −0.2). Women treated with metformin demonstrated less weight gain, 7.2 kg vs 9.0 kg; difference: −1.8 (95% CI: −2.7, −0.9)) and had fewer caesarean deliveries (125 (53%) of 234 in the metformin group vs 148 (63%) of 236 in the placebo group). The infants of the metformin-treated women weighed an average of 219 g less and they were less likely to be extremely large for gestational age and to weigh 4000 g or more at birth (28 (12%) in the metformin group vs 44 (19%) in the placebo group, RR: 0.65 (95% CI: 0.43, 0.99)). Metformin-exposed infants had significantly reduced adiposity measures, mean sum of skinfolds of 16.0 mm (s.d. 5.0) vs 17.4 mm (s.d. 6.2); difference of −1.4 (−2.6, −0.2) and mean neonatal fat mass of 13.2 (s.d. 6.2) vs 14.6 (s.d. 5.0). However, more infants were SGA in the metformin group than in the placebo group. Currently, no data are available that reports the long-term metabolic effects of metformin on offspring health when it is used for type 2 diabetes in pregnancy. A larger (1200 women), randomized, double-blinded, multi-centre clinical trial of insulin plus metformin vs insulin plus placebo for the treatment of type 2 diabetes complicating pregnancy (medical optimisation and management of pregnancies with overt type 2 diabetes study, MOMPOD study) is currently underway in the US (https://clinicaltrials.gov/ct2/show/NCT02932475, accessed 20th August 2021). The primary outcome is a composite adverse neonatal outcome and initial follow-up of neonates until 30 days of age (54).

Pharmacotherapy-targeting obesity within pregnancy outside of the treatment of GDM or type 2 diabetes is not part of routine care. A Cochrane review found insufficient evidence to support the use of metformin for women with obesity in pregnancy for improving maternal and infant outcomes, though acknowledged limited data were available (55). Two included studies have subsequently reported infant outcomes. The effect of metformin on maternal and fetal outcomes in obese pregnant women (EMPOWaR) trial reported body composition, peripheral blood pressure, arterial pulse wave velocity and central haemodynamics in infants exposed to metformin (*n* = 19) or placebo (*n* = 21) *in utero*, at a mean age of 5 years old. No differences were found in any parameters in the children born to mothers with obesity who took metformin vs placebo in pregnancy (56). The metformin in obese non-diabetic pregnant women (MoP) trial followed up 151 children (77 exposed to metformin prenatally) at 3.9 ± 1.0 years. There was no significant difference in peripheral blood pressure, arterial stiffness, metabolic profile and body composition apart from gluteal and tricep circumferences, which were significantly lower in the metformin group. Compared to the placebo group, infants exposed to metformin had significantly lower central hemodynamic function (mean adjusted decrease, −0.707 mmHg for aortic systolic blood pressure, −1.65 mmHg for aortic pulse pressure and −2.68% for augmentation index) and lower left ventricular diastolic function (adjusted difference in left atrial area, −0.525 cm^2^ in isovolumic relaxation time, −0.324 msec and in pulmonary venous systolic wave, 2.97 cm/s) though whether this translates to differences in long-term cardiovascular risk is unknown (57).

### Pre-pregnancy

Despite the identified importance of pre-pregnancy weight management, data on interventions are extremely limited (58). A systematic review of studies of women intent on conceiving, either spontaneously or with assisted reproduction technologies, demonstrated a mean difference in weight loss of >3 kg in favour of lifestyle intervention and an increase in spontaneous pregnancy, though no effect on pregnancy or fetal outcomes was shown (59).

Pharmacological interventions including metformin and appetite suppressing anti-obesity drugs (phentermine, sibutramine and orlistat) combined with lifestyle interventions delivered to women with overweight or obesity in the preconception period demonstrated a weight loss of up to 6–8 kg compared with placebo (60). However, the populations were women seeking fertility and/or affected by PCOS limiting the generalisability of these findings. There are also no data to support any improvements in metabolic parameters of the women during pregnancy or longer term. Further, phentermine and sibutramine are no longer available because of safety concerns.

There is currently no evidence that preconception pharmacological interventions benefit offspring metabolic parameters. Only one study, investigating preconception pharmacological weight-loss interventions vs control in obese women with PCOS, reported on neonatal outcomes. No differences were detected including pregnancy loss, congenital malformations, birth weight or live birth rates, although the study was underpowered for these outcomes (60).

Weight loss medications are not recommended in pregnancy and women are asked to defer pregnancy for 2 years following bariatric surgery given safety concerns. Women who have undergone bariatric surgery have a lower risk of GDM, gestational hypertension, postpartum haemorrhage and rates of caesarean section compared to women matched for their pre-bariatric surgery BMI. However, there is an increased risk of intrauterine growth restriction and preterm delivery, but no difference in stillbirth, malformations or neonatal death (61).

The limited uptake of pre-conception counselling and a lack of clear referral pathways for women at increased metabolic risk in pregnancy are likely to be major factors in the delivery of pre-pregnancy interventions. Recent qualitative work has highlighted key desirable descriptors of interventions in this patient group. Simplicity, flexibility, time-efficient interventions that factor in family commitments and budgetary constraints are seen as desirable (29). Paucity of data relating to efficacy and deliverability is likely to further limit the availability of such pathways to target this patient group.

#### Postpartum and interconception

The postpartum period can be viewed as an opportunity for interventions targeting long-term morbidity, as well as interventions aimed at minimising metabolic risk in subsequent pregnancies. Public Health England identifies the 6–8 week postnatal check as an opportunity to discuss the mother’s weight and to determine whether she would like any advice or support now, or in the future, as well as recommending that women with a BMI of >30 kg/m^2^ should be offered referral to a structured weight management programme (62). A systematic review of 36 randomised–controlled trials including 5315 women involving lifestyle modification interventions in post-partum women (up to 2 years after birth) (63) found participation rates were typically 20–40%. From the 23 studies that provided sufficient information, the pooled mean weight difference (95% CI) was −2.33 (−3.10 to −1.56 kg) (63). There was no reported data of any effect on maternal or offspring outcomes in subsequent pregnancies. Implementation research may need to focus on the most at-risk groups. In particular, ethnicity, deprivation and education status are all associated with increased weight retention at 1-year post-pregnancy (64). Lifestyle interventions have been the major focus of previous research, though there may be a role for medication in some patient groups. It is important to note that weight loss medications including Saxenda and Orlistat are not licensed for use in breast feeding and must be discontinued in advance of considering further conception. Diabetes treatments for this patient group who may be considering further pregnancies are likely to be limited to insulin and metformin unless effective contraception and patient education are assured (65).

One of the key barriers to metabolic health in the post-partum period is the low uptake (<20%) of screening for type 2 diabetes in women with previous GDM (36). Lifelong screening to detect onset of impaired glucose tolerance/type 2 diabetes is recommended for women who have had a pregnancy complicated by GDM. However, regular screening for other cardiovascular risks factors such as hypertension and hyperlipidaemia do not feature in international recommendations, demonstrating a failure to recognise the wider cardiometabolic health risks of this patient group (27, 28). Given that type 2 diabetes can largely be prevented or at least delayed by lifestyle intervention and loss of bodyweight (66), annual screening and follow-up in primary care for women with GDM and their offspring must be prioritised as a part of population health prevention programmes. Working partnerships among primary care healthcare professionals and holding education events aimed at healthcare providers have been shown to significantly improve screening rates (67). Significant work is in progress to develop prediction models for type 2 diabetes following GDM (68). Such work may allow targeted interventions aimed at prevention, as well as increased frequency of monitoring in those most at risk. Interventions aimed at reducing type 2 diabetes risk in this patient group include diet, physical activity and medications. Delivery of any interventions will need to be carefully planned given the constraints on time of this patient group who often have a new baby and may be returning to employment alongside other commitments. An ongoing RCT examining the impact of a blended approach of telephone and in person contact is likely to be informative (69). A systematic review and meta-analysis of 11 RCTs of diet and physical interventions post pregnancy in women with prior GDM studied the effect on type 2 diabetes risk. The duration of these interventions was between 4 months and 3 years. Of the ten RCTs starting the intervention within 3 years post-partum (*n* = 1733), there was a pooled relative risk of long-term type 2 diabetes of 0.57 (95% CI: 0.42–0.7) (70). Following the ESTEEM study (46) on Mediterranean diet in pregnancy, there is now a feasibility randomised control trial, using Mediterranean diet in the post-partum in women with prior GDM (Mediterranean diet to prevent type 2 diabetes in mothers who had diabetes in pregnancy: a feasibility study, MERIT) (https://www.isrctn.com/ISRCTN40582975, accessed 20th August 2021).

The role of medications in this patient group is of increasing interest. A feasibility study is currently in progress studying the effect of metformin on type 2 diabetes risk in women with GDM with the primary outcomes of recruitment, randomisation, adherence and attrition and secondary outcomes of glycaemia, cost and quality of life (Optimising health outcomes with Metformin to prevent diAbetes After pregnancy, OMAhA) (https://www.isrctn.com/ISRCTN20930880, accessed 20th August 2021).

**Figure 2:**
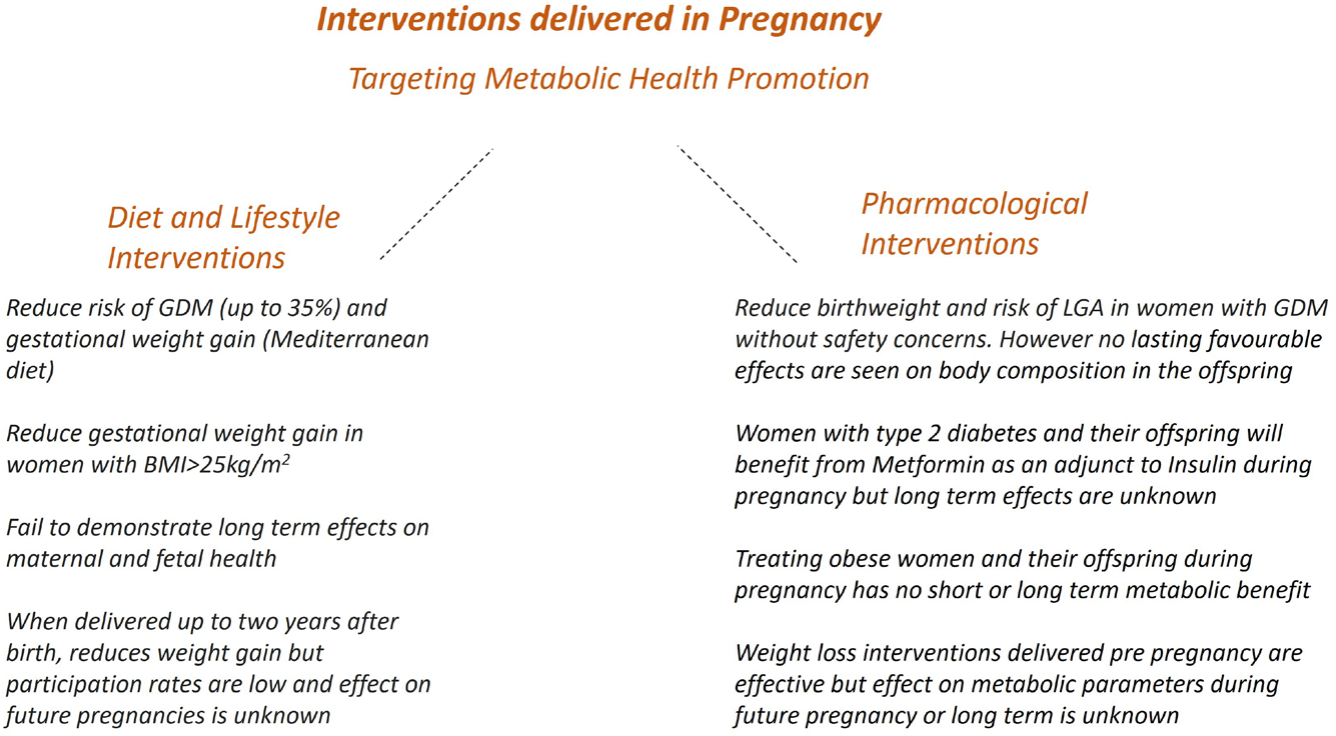
Summary of interventions across the pre-, intra- and post- pregnancy periods, targeting metabolic health promotion.

There is a considerable amount of research and clinical impetus in promoting maternal and offspring metabolic health through interventions delivered across the pre-, intra- and post- pregnancy periods, see Fig. 2 for a summary of research findings. Collaborations between international researchers in this area, patient groups and clinicians continue with the aim of improving maternal and offspring health.

## Implication for practice during and post pandemic

Evidence demonstrates women with poor metabolic health are at increased risk of severe COVID-19 infection during pregnancy. PregCOV-19 a living systematic review and meta-analysis of COVID-19 infection in pregnancy identified 192 studies, including a total of 64 676 pregnant women and 569 987 non-pregnant women with COVID-19 infection. The odds of admission to the intensive care unit and need for invasive ventilation were higher in pregnant and recently pregnant women with COVID-19 compared with non-pregnant women of reproductive age. Pre-existing comorbidities, non-white ethnicity, chronic hypertension, pre-existing diabetes, high maternal age and high BMI were risk factors for severe COVID-19 in pregnancy (71).

At the start of the pandemic, radical changes were made to the delivery of antenatal services to minimise contact and reduce spread of infection. Pregnant women were encouraged to shield or strictly follow social distancing measures by staying at home. Pre-pregnancy counselling services were halted, and women were encouraged to continue effective contraception. In high-income settings, new GDM care pathways were adopted with changes in the choice of biochemical tests (increasing use of HbA1c and random plasma glucose rather than OGTT) and glucose thresholds used for both screening and diagnosis of GDM, as well as reducing face-to-face consultations with the multidisciplinary team by introduction of telemedicine clinics for remote education and monitoring of glycaemic control (https://www.rcog.org.uk/globalassets/documents/guidelines/2020-12-09-guidance-for-maternal-medicine-services-in-the-coronavirus-covid-19-pandemic.pdf) (https://www.adips.org/documents/RevisedGDMCOVID-19GuidelineFINAL30April2020pdf_000.pdf, accessed 23rd July 2021), https://www.adips.org/documents/RevisedGDMCOVID-19GuidelineFINAL30April2020pdf_000.pdf, accessed 23rd July 2021), (https://els-jbs-prod-cdn.jbs.elsevierhealth.com/pb/assets/raw/Health%20Advance/journals/jcjd/JCJD_COVID_guidelines_020420-1585856697530.pdf, accessed 23^rd^ July 2021). Data suggest that alternative tests to OGTT including fasting plasma glucose and Hba1c have lower sensitivity for diagnosing GDM, but specificity may be sufficient to safely identify those women at highest risk (72). There is an urgent need to review the impact of these changes in diabetic care pathways on maternal and neonatal outcomes. Findings will facilitate planning care delivery for women with diabetes in pregnancy as we emerge from this pandemic.

Use of technology to deliver antenatal healthcare remotely had been gaining increasing popularity prior to the pandemic, particularly for the management of GDM. Interventions include telemedicine clinics and use of smart phone apps for remote monitoring of glycaemic control. A meta-analysis of 32 RCTs with a total of 5108 patients showed that the telemedicine group had significant improvements in HbA1c, MD −0.70 (95% CI: −1.05, −0.34), fasting blood glucose, MD −0.52 (95% CI: −0.81, −0.24) and 2-h postprandial blood glucose, MD −1.03 (95% CI: −1.83, −0.23) compared to the corresponding parameters in the standard care group (73). Maternal and neonatal outcomes were also improved in the telemedicine group with significantly lower incidences of caesarean section RR 0.82 (95% CI: 0.69, 0.97), neonatal hypoglycaemia RR 0.67 (95% CI: 0.51, 0.87), premature rupture of membranes RR 0.61 (95% CI: 0.50, 0.76), macrosomia RR 0.49 (95% CI: 0.30, 0.80), pregnancy-induced hypertension or preeclampsia RR 0.48 (95% CI: 0.40, 0.58) , preterm birth RR 0.27 (95% CI: 0.20, 0.35), neonatal asphyxia RR 0.17 (95% CI: 0.08, 0.33) and polyhydramnios RR 0.16 (95% CI: 0.10, 0.28) (73). Similar interventions have been tested in the management of obesity in pregnancy. A study comparing telehealth to deliver lifestyle interventions with standard antenatal care showed a modest impact on gestational weight gain without impact on perinatal complications (74). Though this has advantages for certain patient groups, there remains a risk that it could further health inequalities and disproportionately affect non-native speakers or those from more deprived backgrounds who may have limited access to technology ‘Digital poverty’.

## Research recommendations

The identification of and personalisation of risk are likely to be a key component of future healthcare. To better identify women at risk, we need to develop robust, individualised composite risk scores. Such scoring systems need to be developed across pregnancy for the preconception, early pregnancy and postnatal periods and take into consideration personal, familial and pregnancy-specific factors. The development of capable tool(s) will require the use of machine-learning algorithms and will be reliant on access to and sharing of large-scale clean data sets with tens of thousands of data points, where pregnancy records are linked to general health records and to those of the offspring (75). The role of risk prediction of GDM is relevant across pregnancy with current selective screening strategies failing to identify large numbers of patients with GDM. Furthermore, such screening strategies should incorporate low- and middle-income countries which contribute 90% of the world’s cases of GDM (76). Current screening strategies also miss GDM early in the pregnancy and the impact of early identification and treatment of GDM in the first trimester should be a subject of investigation.

Women with established GDM are also likely to benefit from more targeted risk in both early and late pregnancy both to allow the differentiation of low and high-risk pregnancies affected by GDM and to identify an individual’s long-term risk of type 2 diabetes more accurately. Stratification of risk of both complications within pregnancy and longer-term metabolic risk might more easily allow for targeted interventions being delivered at risk groups and allow the de-medicalisation of low-risk patients (77).

While the identification and quantification of risk are clinically useful to better inform the patient and clinician alike, it must happen in parallel with evidence-based interventions. Traditional interventional studies have targeted whole populations. Moving away from this approach to precision-based treatment aligns with ongoing research on personalised risk stratification and understanding of the GDM phenotype. Such interventional studies are likely to involve large multi-centre studies with clearly designed interventions informed by systematic reviews in this area (78). They should include long-term follow-up of both the mother and offspring to allow for clear recommendations around care to be made as well as accurate estimations of health economic and societal impact. Given the complexities of such studies and the challenges of long-term follow-up, these studies are likely to rely on integrated access to routinely collected clinical data for both the mother and the offspring. Though large-scale studies are required to inform clinical practice, smaller studies with a greater focus on implementation research are required in conjunction to ensure that marginalised patient groups are able to access and receive high quality care.

## Conclusions

The spectrum of metabolic dysfunction including obesity, GDM, type 2 diabetes and PCOS is increasingly common in women of child-bearing age and the prevalence of metabolic dysfunction seen in pregnancy has doubled within the last decade (1). Obesity is a major determinant of long-term metabolic ill-health in both the mother and the offspring (9, 11, 12, 79). In addition, the risks of GDM within pregnancy on maternal and neonatal outcomes are well established (10, 13, 79). Co-existent obesity and GDM further exacerbate these risks. PCOS is an independent risk factor for maternal and neonatal adverse outcomes (22). The role of PCOS on the long-term metabolic risk of the offspring is less certain though the combination of PCOS and obesity seems to confer significant risk (80). Pre-existing diabetes irrespective of type is associated with complication risks to mother and offspring with higher HbA1c, deprivation and type 2 diabetes, a particular risk (6). Pre-existing maternal diabetes and obesity are well-established risk factor for premature cardiovascular disease in the offspring as young adults (12, 81).

Despite the importance of identifying poor maternal health and offering treatment prior to and following conception (82), the available interventions and their impact are limited with only a modest effect on gestational weight gain (44) and difficulty following up women post-delivery. There has been a significant research effort targeting women across their pregnancy. However, the limited availability of services to improve metabolic health especially in the pre and post-pregnancy period is a significant limitation in the realisation of improved clinical care. Clinical care pathways aimed to support more marginalised patient groups will be required to ensure equitable access to care and to improve both short-term and long-term metabolic ill-health both within the mother and offspring.

## Declaration of interest

The authors declare that there is no conflict of interest that could be perceived as prejudicing the impartiality of this review.

## Funding

This work did not receive any specific grant from any funding agency in the public, commercial or not-for-profit sector.

## Author contribution statement

This manuscript was prepared by Dr Niamh Mclennan and Dr Jonathan Hazlehurst. Prof. Reynolds and Prof. Thangaratinam agreed the overall content, edited the manuscript and approved the final draft for submission. S Thangaratinam and R M Reynolds: joint senior author.
